# Tracing Historical Forms of Servitude: Introductory Remarks and Elementary Reflections

**DOI:** 10.1080/19472498.2022.2050025

**Published:** 2022-03-15

**Authors:** Nitin Sinha, Pankaj Jha

**Affiliations:** a Leibniz-Zentrum Moderner Orient, Berlin, Germany; b Department of History, University of Delhi, New Delhi, India

**Keywords:** Domestic servant, domestic service, South Asian history

## Abstract

History of domestic service in South Asia is beginning to attract scholars but a rich historiography around it is still distant and difficult. The forms of servitude are different across different times, places and contexts. The sources available to scholars for specific sites and moments in history are marked by extreme diversity of language, genre, concerns, and vantage points. Studies based on exploration of particular kinds of texts across dissimilar contexts make for a good beginning. Using insights from apparently disjointed explorations of servitude in uneven locales, we sketch a tentative template to study changing patterns of service relations and their articulations in the long duration beginning with the early modern period and reaching up to our own times. The study of domestic service yields empathetic vignettes of lives of domestic servants. They also yield insights on various forms of servitude and their centrality to social and economic relationships in a manner that can potentially upset the set historiographic paradigms of work, leisure, household, labour and ‘labour laws’ in the early modern and modern periods.

In the critically acclaimed 2019 Hindi movie Thappad [A Slap], the upper middle-class protagonist Amrita painfully discovers her servitude within an ostensibly caring marital relationship only after her husband publicly slaps her in an apparently ‘exceptional’ moment of exasperation. A chilling scene in the film comes in the morning after when Amrita shares the frame, if not the fate, with her maid servant Sunita who, she knows, is regularly assaulted by her own husband. Amrita awkwardly avoids the maid’s gaze, while the latter appears eager to offer her quiet empathy. Why does the mistress not look at the maid’s diffident quest for solidarity as a source of strength? Why does the thought of cementing a common, even a non-verbal, bond out of marital violence make her uneasy?

It is difficult to explain this reluctance only in terms of a functional class divide that exists between masters/mistresses and servants, or to use the modern terms of reference, between madams/sirs and their ‘helps’. Like any other abiding relation of asymmetrical power, the relationship between master/mistress and servants too carries multiple burdens of centuries old complex history. The immediate context of this relationship as shaped through work interlaces with historical fault lines of power, especially those of caste, class, gender, and other types of hierarchies that operate at the broader social level. Added to this is the uniqueness of domestic service, which lies suspended in the tension running across the two ends of a spectrum. At one end is the notion of the space of work, which is the same as the domestic (the household including its innermost private or ‘pure’ parts). In such a case, the masters and the servants, the employers and employees, find themselves in a proximate, intimate bind without in anyway diluting the exploitative and hierarchical character of the bind. There is a certain amount of conviction to treat the household as a sanctimonious place, which should not be treated as a workplace.^1^ In the last 100 years or so (but also stretching back further in time) on the other hand, the second end of the spectrum has strongly come to be tied to the wage-based form of labour market of domestics where the maid or a male servant is also a ‘worker’, within a transactional relationship based upon relative clarity of terms and conditions of hiring.^2^ This combination of work- and space-based proximity on the one hand and the ‘distant’ transactional mode of employment/occupation together with the social faultlines of identities and institutions on the other, engender a non-linear spectrum of very complex forms of servitude before us. Even in the apparently benign change in forms of address and identification – from servant to employee to the more recent ‘domestic help’ – a deeper dynamic of change and continuity of historical forces are at work. Servants came to be regarded as employees broadly as a result of the legal changes in terms of contract that defined the modern form of employment in the second half of the nineteenth century.^3^ This indicates an interlinked history over a longer temporal span from that of the colonial moment to the present: from being a subject of regulation (under the master-servant regulations) then to falling outside the domain of regulation (that is, labour or social security laws) now.^4^ Yet the ‘feudal’ imageries of subordination co-exist with modern forms of employment in the postcolonial phase.^5^

As scholars of South Asia, however, we are still only beginning to explore this trajectory of change and continuities. For, paid domestic service and work has received scant scholarly attention in South Asian history writing. We indeed have a gradually growing number of researches, mostly on the contemporary period, on two related aspects of domestic work. One of these revolves around the ‘anxieties’ and predicaments of informality, which defines the current nature of domestic work. The other concerns legal protectionism, which has pushed civic and non-governmental organizations to take up the issue towards legal formalism.^6^ Historical genealogy of domestic work, on the other hand, has attracted scant scholarly attention.^7^ This is one reason why we are still ill-equipped to fully understand what layers of historical debris might pull down or push up specific aspects of the master-servant relationships in particular contexts. It was the burden of history that played out in that moment of encounter in the movie, when the possibility of forging a gender-based solidarity unsettles Amrita, and the proximate difference based on class triumphed over her thought process. The only trace the burden of history leaves in the film is the lingering awkwardness of the moment.

The challenge before scholars is to attain a balance in describing and analysing the structures that have produced and sustained the ubiquity of domestic work in South Asian households with the ways in which it operates at the everyday level of social- and work-based relationship. This is not possible without paying close attention to, among others, registers of language, variations in regional cultures and practices, and temporal and ideological shifts in practices of law and governance that have accompanied the long history of (paid) domestic work. The interchangeable use of service, work, and servitude adds to the depth of the challenge facing probable historiographies of servants and servitude, long ignored in the many subaltern narratives of the social and political histories of South Asia. The eight essays in this special issue spanning the last 400 years attempt to achieve this. They do so with reference to specific textual materials, temporal contexts, and historical contingencies, and without falling for the temptation to make easy pan-India generalizations.

## I

Servants are ubiquitous in cinema, literature, and real life in historical and contemporary South Asia. Yet, they have been curiously absent from scholarly discourse until very recently. A recent work in two volumes involving a number of early-career and established scholars (including both the editors of this special issue) is among the very few and the first major work that have begun to undertake an elementary and consolidated exploration of the histories of servitudes in a variety of times, places, and contexts.^8^ A detailed discussion on why the servants’ pasts rarely attracted serious scholarly attention together with the ‘review’ of the literature existing on this theme was undertaken in the long introductions to those volumes.^9^ We hope that our assertions and expostulations in those volumes would be read and critiqued/contested widely and meaningfully to enrich our understanding of the historiographic lacunae. As for the rationale behind this volume as a separate collection, there are many. One of its distinctive features, in comparison to the two published volumes, is a set of three essays that exclusively deals with Urdu printed materials and household literature of the nineteenth and twentieth centuries. In the second published volume (dealing with the post 1800 period), we had tried to understand the ‘social’ realm of domestic service in the literary representations of the colonial and postcolonial period largely through Hindi printed materials alone. Of comparable merit is the intense and expanded focus on the contemporary social and political conditions in which domestic service is provided and ‘regulated’. Secondly, in the sphere of the early modern period also, this volume moves forward from our earlier focus on normative and structural conditions (for instance, the role of *akhlaq* and caste norms) to actual historical studies of certain groups that were positioned in the complex relationship of service and servitude. Notwithstanding this widening of the scope and the persistent attempt to create a dialogue between the past and the present, suffice it to say here that one of the challenges for historians at this stage is to grapple with the wide variety of sites, sources, and temporalities within which servants as well as different types and cultures of servitudes are/were located. It is not possible to reduce different relations of servitude to a few classified types or typologies. And yet often, as an element of methodological practice of organizing research, these categories appear to become the first entry points whose unmasking lays bare the ‘hidden scripts’ of intimacy, resistance, and the everydayness of the unequal relationships. In this regard, it is equally important to let categories bounce back to and jostle with each other, for instance, of slaves and servants to capture the interstitial spaces that are usually saturated with ‘normalized’ norms and forms of interactions that happen between masters/mistresses and servants.

In South Asia, for example, every language (of the hundreds that have survived), probably has at least two, if not more, words for servants. Yet, these words are not precise synonyms of each other, embedded as they are in unique and specific contexts. Some times the same word, in a different form, may signify a distinct relation of asymmetry. Thus, for example, the most common north Indian word for servant, *naukar* carries a pejorative sense. A common refrain one makes in north India, in the face of unreasonable demands from someone is, ‘*naukar samajh rakha hai kya*?’ [Dare you consider me your servant?]. Yet, the word *naukari* [literally what the naukar does] has come to signify respectable employment, indeed any employment. Even the most qualified unemployed youth in north India aspires for ‘naukari’! Equally significant is the complex historical trajectories that these words have moved through. As Oesterheld shows in her piece in this special issue, naukar was not always a pejorative term. Even in the nineteenth century, the word could be used for anyone ‘employed’ gainfully by an institution or person without signalling disrespect or low status for her/him.^10^ In a similar vein, in English, servant and service might be cognate words, but they connote qualitatively very different sets of relationship. No less confounding is the fact that many relations of servitude exist behind the veneer of another, usually a more respectable tie, or as a major, if not a defining, component of such ties. In several real, literary, or cinematic cases, it is easy to see a prominent component of unpaid, abject, and ‘glorified’ servitude in motherhood as well as in the figure of a virtuous wife or dutiful daughter-in-law. Conversely, empowering and emancipatory relations would sometimes strategically deploy the epithet of servitude (even slavery) to god to cut themselves loose from certain worldly burdens. Such indeed was the case with those bhaktas of the early modern world, who often wilfully took the suffix of ‘das’ (slave) in their names. The pathway of emancipation was figuratively carved through the burden and (self) imposition of bondage. The same may also be said about political leaders in the electoral democracies all over the world, (but nowhere more than in postcolonial India) who love to call themselves ‘servants of people’. The acquisition of these epithets as an agentic act (and to an extent symbolic as well) to make moral or pious claims is of course different from our concern of unravelling the histories of those who were structurally positioned in the social hierarchy to provide service either as a form of employment or as an outcome of social relations. Thus, while the scope of the term ‘servant’ or even ‘domestic servant’ is wide enough to embrace an array of hierarchical positions in which some might appear less of a servant than the other, the heuristic valence of this term is in regarding it as a collective – akin to other terms such as peasants and workers – as well as in analytically excavating the many meanings and worlds of work and status enfolded therein.

Historians trying to explore the variegated histories of servants and servitudes in pre-modern, early modern, and modern South Asia have to navigate through these entangled representations and multilayered contexts. The present collection of essays is another attempt at a foray in this recently opened field. Taken as a whole, the temporal span of the papers lies between the sixteenth century and the contemporary period, with one of the papers (Lokesh) focusing on a micro-study of a ‘riot’ in 2017. However, this compendium does not claim to be a comprehensive coverage of the period. It is not even a survey of change and continuity in relations of servitude all over the subcontinent or a major part thereof in the said period. Indeed, readers of the journal might be forgiven for thinking, in the first go, that these are relatively disconnected spheres and themes with little in common. Yet a closer look at them might reveal similar but novel ways in which our contributions re-read certain historical sources to excavate the history of the servants. New ethnographies on the other hand have been purposely conducted to understand the notions of caste, gender, and work in cities like Kolkata and Noida. Finally, a novel theoretical approach has been adopted to understand the exclusion of domestic servants from the corpus of labour rights, and in doing so, to also understand the role of the state in the postcolonial period.

The papers by Neha Vermani and Lubna Irfan are set in the Mughal period and engage with textual and visual materials. Moving beyond the usual refrain of statecraft, mercantilism, trade, and the recent spurt of writings on literary cultures of early modern period, these two papers focus on social groups of service providers thereby ‘scaling down’ the lens through which much of the Mughal history has hitherto been written. Essays by Christina Oesterheld, Ufaque Paiker, and Jamal Ali Magsi focus on the erstwhile aristocratic (and ‘normal’) well-to-do ‘Muslim’ households in the nineteenth and the twentieth centuries. All three of them base their research on close readings of certain Urdu texts. Between the three of them, they cover genres as varied as, household manuals, didactic texts, poetry, personal letters/petitions, and (auto)biographies of Muslim literati of the times they are dealing with. They reveal a fascinating array of ways in which nature and notion of households change during the period revealing also how the servants’ role within those households were rewritten. In the last three by Sonal Sharma, Lauren Wilks, and Lokesh, dealing with postcolonial India, we encounter the narratives of legal practices of the state among other things. They also explore where the state and its agencies stand vis-à-vis the absence of domestic workers in protective labour regulations. What emerges is a rich ethnography-based account of both the mundane and the episodic character of the relationship between upper middle-class lords/mistresses (employers?) and domestics (employees?) in urban settings of India.

It is equally important, however, to take each paper in its own right and in its own specific context. Their authors were charged with no uniformity of methodological choices or even a fixed set of questions. The common concern that might probably have guided them was to unearth what might appear to each of them as the most interesting aspects of the master-servant relationships given the scope, limitations, and nature of the sources they were dealing with. In this brief introduction, however, we will take the liberty of highlighting the broad shifts which we can map on the basis of the findings presented in these papers.

## II

The first two papers of the special issue are set in the Mughal period. Lubna Irfan explores servants’ varied roles in the noble and royal households. She looks at their daily chores in the household, their work in the kitchen, stables, and in the *karkhanas* (workshops) as well as their roles as musicians. She outlines and analyses shades of exploitation within the existing fault lines of gender, especially in the context of the widespread deployment of eunuchs (*khwajasaras*). The liminal socio-sexual identity of *khwajasaras* throws up interesting vignettes of service conditions and stretch our imagination about what was possible and what was not within an unequal relationship that resided within the liminal space of legal slavery and other forms of bondage. This becomes more evident in her treatment of other two categories of *chelas* and *sahelis*, who were free in principle but whose services were not very different from those of the slaves owing to the ethical, moral, and structural context of ‘complete submission’ within which they functioned.^11^ The question at stake here, we believe, is not to concretely ascertain, juridically or otherwise, who was a slave or a servant (based upon the logic of free and unfree) but to gauge the elements of coded and felt norms of hierarchical relationship of service that inevitably sailed/glided between the two extremes of ‘free’ and ‘unfree’. Although the paper draws on many different sources, including memoirs and travelogues, the mainstay of analysis is a Persian biographical compendium entitled *Zakhiratul Khwanin* composed by Farid Bhakkari and on the Mughal miniature paintings.

In a related but distinct manner, Neha Vermani also explores the Mughal royal household but her focus is primarily on the kitchen and the dining hall of the palace. While Irfan locates the nature of service and servitude in the logic of Mughal sovereignty, Vermani takes us to the everyday aspect of food that was regarded as a very important conduit for the fashioning of the self. The knowledge and skill that was required to cook and serve, as well as the beliefs about food as an important determinant of one’s personhood made the ‘kitchen labour’ an important work in the physiological and psychological constitution of the kingly and other royal persona.^12^ The strength of her work lies in the manner in which she explores the wilful spatial arrangements in the architecture of the palace, wherein the location of the kitchen and the harem quarters vis-à-vis the rest of the palace and vis-à-vis the distance from the person of the emperor was of crucial significance. This particular kind of spatial arrangement, she shows, had crucial implications for the gendering of the services. Equally important is to decipher the ritual meaning of objects such as *paan* in which the meanings of proximity, privilege, and patronage were embedded.^13^ When the imperial betel stock was misappropriated, Shah Jahan reprimanded the in-charge of the stock because it meant insubordination of the ritual authority of the monarch. Vermani carefully explores both textual and visual materials to see how architecture and objects constituted the broad patterns of master-servant relationship. This is particularly significant in view of the fact that both animate and inanimate objects were seen and valued with reference to the degree of physical proximity with the emperor. Thus, in these two papers, we find a wide range of aspects, from sovereignty to everyday objects, playing a crucial role in defining the everydayness of the service relationship.

The three papers by Oesterheld, Paiker, and Magsi are located in the period between the mid-nineteenth and the mid-twentieth centuries. They analyse north Indian Urdu literature of the time to produce very poignant, and occasionally counter-intuitive vignettes of servitude depicted both in didactic and in descriptive mould. Oesterheld explores a wide range of Urdu literary genres and sources beginning with interesting details from Ghalib’s letters (mid-nineteenth century) and coming up to the early 1900s with a very useful engagement with a guidebook for brides entitled *Bihisthtī Zevar* (Heavenly Ornaments). She seeks to outline patterns of change in normative and descriptive depictions of servants fully cognizant of the genre differences between sources as well as the changing material conditions of the Muslim households for, and by whom, the texts were written.

Two points are important to be mentioned here: first, until the mid-nineteenth century, one can detect the persistence of older norms of trust and loyalty. This is reflected in Ghalib’s letters (Oesterheld) as much as it was true for Shad Azimabadi who reminisced about the role of servants in his writings through the prism of late Mughal high culture (Paiker). The relationship between masters and servants was largely seen as inhabiting the domain of the ‘personal’ (the person in ‘personal’ being that of the master, and not of the servant), and idealized accounts would frequently portray servants as faithful and masters as compassionate. This idealization was further supported by the use of Islamic scriptures and books on *adab* (etiquette) and akhlaq (ethics). But in spite of the (master’s) claims of treating them as members of the family, hierarchies had to be maintained as reflected in the nature of greetings: in Ghalib’s letters written to others, the servants would offer their *bandagi* to the addressees and his adopted grandsons would offer ‘adaab’.

The second point is related to the transitions in the late nineteenth century with the emergence of a middle class. The reform literature of this period, as Oesterheld argues, gave rise to a sharper portrayal of servants as disloyal and treacherous subjects who needed to be more strictly supervised and controlled. These were the households of average means usually employing one to two servants in which the accent on maintaining respectability was given prominence. One significant way of doing that was by maintaining distance from ‘lowly people’ and servants. Indeed, based upon Urdu and Hindi literary writings of this period, an argument, with some speculation, can be pushed further that servants got ‘dragged’ into the discourse of domesticity – mostly through their negative portrayal – not because of their own doing but because the middle-class’s sense of respectability itself was at stake that needed a new gloss that was derived from the twin claims of (perceived elements of) the traditional and the modern. As servants were written into the script of middle-class reformism, their role and characteristics were also constructed and generalized – through stereotypes related to caste and character – rendering them marginal and invisible (or visible only through negation) as the relationship between the master and servant itself became incrementally transactional.

Magsi’s paper moves up the timeline with a focus primarily on the works of Deputy Nazir Ahmed (late-nineteenth century) and those of Ismat Chughtai (going up to mid-twentieth century). As Magsi notes, the servants in their writings were depicted both as a corrupting source for young children as well as a means to patrol the boundary of the permissible for them. (It goes without saying that in the texts authored by and for the ‘servant keeping classes’, ‘children’ never referred to the servants’ own children). In fact, Magsi in some ways complements the descriptive picture sketched by Oesterheld in forcefully arguing that it was the ‘servant problem’ in the core of why Nazir Ahmed turned his gaze towards talking about reforming the households. In his didactic tales, as Magsi argues, Ahmed persistently brought servants to the fore; the focus, as widely discussed, is not only on the education of young girls but also on patrolling the boundary of interaction with the servant.

Taken together, these two papers track the changes emerging from *nawabi* to that of *sharif* households. Magsi marks out the generational shift as well. In her stories, Ismat Chughtai identified and assessed the exploitative relationship within which servants were condemned to live. Under the progressive impulse of Chughtai, the master-servant relationship becomes the template to understand the nature of class inequalities. Magsi’s analysis of her stories shows how Chughtai used the figure of domestic servants to highlight the hypocrisies and contradictions within middle-class Muslim households whose ‘ideal’ conditions were sketched a generation earlier by Ahmed. In her stories, servants appear as talking, thinking, and acting subjects in contrast to just being the ‘object’ of compassion and control. A further extension of the scale at which the master-servant trope operated is shown in those of her stories in which servants disrupt the narrative of nationalism. Servants’ notion of ‘unbelonging’ to the nation brings forward the structural differentiation between the privileged and the deprived, which is derived from and based upon the essentialized yet contradiction-ridden relationship between masters/mistresses and servants.

Such a combination of the structural and the everyday contradictions is beautifully explored by Paiker through the case study of a decaying nawabi style household of Patna in the late nineteenth and early twentieth centuries. One of the features of the transition from nawabi to sharif modes of household organization, as outlined by Oesterheld, was the sanitization of the sexualities of female domestics, or of sexual liaisons between erstwhile nawabi male householders and the female domestics of the household. This depiction was now pushed, as she argues in her essay, to the ‘racier commercial literature of the late nineteenth century’. Paiker’s entry point is the poem written by Shad Azimabadi on his *maalan* (female sweeper) with whom he had intimate relations. This became a mocking point in the Urdu literati of the city. The contention over the poem, as Paiker argues, denotes the larger contestation over the meaning of *ashraf* and the role (as well as the ideal form) of the master-servant relationship therein. In the earlier times, as she argues, the relationship was couched in terms of adab tradition but by the end of the nineteenth century it came to be fashioned by conventions of caste (*zat*). Reading the history of the household first through the autobiography written by Shad and then Shad’s biography written by his grandson, Paiker enumerates the changes such as the reduction in the number of servants, form of payment, and spatial alignment in the position of servants. Locating the household at the cusp of the changing political economy (which saw the expansion in wage-based labour market as well as contraction of pension-based aristocratic households), she also ties this with the ideological shift from adab to caste of which, among others, a chief aspect was the recasting of servants as devious and dishonest, given to stealing.

The five papers covering the period from the sixteenth to the early twentieth centuries, map several related ideological shifts: the reconstitution of the households’ norms, the gendered and spatial segregations that existed within the household but also in a city that could impact the service relationships; and changes in the nature of sovereignty and political economy. There are indeed broad-brush surveys and tentative patterns presented in these essays, but equally, these patterns are rooted in the history of specific groups, or case-studies of households. Inevitably, there remain gaps as well which hopefully the future researchers will detect and bridge.

On the margins of the mainstreamed view of servants as deviants and objects of control (that took strong roots through the popularization of print and reform literature on the household by the early decades of the twentieth century), there also existed on the ‘fringe’ a more radical view in literature that tried to capture the unspeakable pathos of servants.^14^ In the area of statecraft, from the late colonial phase to the early postcolonial, the associational politics of ‘labour’ had become widespread. In the 1950s, there emerged a new language and new politics around the question of labour. Yet domestic servants were kept outside the legal protective web, thus denying them the identity of a ‘worker’, a practice that continues till date. This failure to incorporate servants into the fold of labour, as Sharma shows, was achieved by invoking deep historical entanglements of service with issues of caste and poverty. It was argued that the absolute contractual frame, if imposed through law, would dismantle the old paternalist structure, which the opponents tied with the glory of the past and tradition. The idea of the family and kinship was used to secure a ‘good’ relationship between employer and employee rather than allowing ‘modernity and industrialism’ to shape the ‘private’ relationship within the household. The privatization of the relationship was thus a historically produced setting, chiefly through exclusion by design. Between the 1950s and the 1980s, rather than recognizing domestic service as work, the issue was, as Sharma shows, subsumed under the category of ‘poverty’ arguing that once poverty declines in India, domestic work will also cease to exist. From the 1980s, when demand for better regulation of domestic work started to appear, once again, the issue was not directly addressed and was crushed under other cognate concerns on child labour and human trafficking. While the state has in the last 10 years or so at least recognized the need for protective regulation, the language of skill has recently emerged, which might drown the fair demands to regularize work relationship between employers and employees.

The effect of this new bureaucratic language of skill on the service relationship is yet to be seen. Modern discourse on the domestic service has often vacillated between the competing languages of ‘love’ and contract. Wilks, through her rich ethnography of Calcutta households, proposes to understand this contest in which servants navigate through the framework of ‘pragmatic intimacy,’ which she borrows from Sen and Sengupta’s work.^15^ Wilks’ essay captures the everyday routine of work through food, drinks, ‘gift’ (usually of discarded materials), access to toilets, and ideas of hygiene, which reveal that intimacy is invoked strategically from both sides for respective benefits and comfort. Two crucial elements emerge from Wilks’ ethnography, which are worth highlighting here. First, although caste, gender, and class has historically played a significant role in shaping the master-servant relationship, there is a growing belief amongst domestic servants that ‘work’ is central to this relationship. They define their work in terms of ‘kaaj’ (Bengali word for work) rather than service. The centrality of work in self-identification has also become crucial because these urban usually part-time commuting maids are well aware that it is their quality of work that eventually matters in their employment otherwise the employers won’t even recognize them on the streets once out of the service.^16^ This notion of work encompasses also the element of continued/uninterrupted service as taking leave is risky for workers in the context of hyper-insecurity.

The assertion through the identity of work might be pushing the servants, as a recent work has pointed out, to ‘challenge “ideals” of silence, passivity and invisibility’.^17^ That the perceived nature of work – polluting or non-polluting, broadly distinguished between kitchen work and bathroom cleaning work, respectively, – is also a concern for servants and not only employers.^18^ The second point which Wilks stresses is the role of time in this new set-up of work. These workers work in multiple households and each household attempts to maximize the use of labour in that limited time. Workers themselves suffer the constraints of time due to long hours of commuting which plays a role in making their jobs further insecure. As commuting workers, like migrant workers, are often willing to work for lower wages than city-dwelling workers, and as they work in different households amidst stiff competition for work, a peculiar characteristic has emerged: they are most autonomous and most insecure of the domestic workers.

Finally, wading through the failed attempts of legislative protection and routinized nature of work constituted through pragmatic intimacy and quotidian contestation, we arrive at the most episodic moment of conflict in the recent past, which happened in a high-rise urban dwelling of Noida in 2017. Rather than knowing which side was actually at wrong, Lokesh through her ethnographic study, conducted with difficulty due to people’s reluctance to share details, uncovers the politics of service as emerging in the ‘new India’ of gated high-rise communities. It is a micro-study of a ‘riot’ in which employers, employees, and the state participated. However, the event is explored not exactly in the Ginzbergian sense of ‘microhistory’ which involves undoing the seams of the narrative – usually judicial – to bare the inconsistencies of ‘truth’. Rather, the author uses the event of the riot as an entry-point to understand its making, it’s possible repercussions on lives and livelihoods of servants, and what it represents in terms of structural and spatial organization of work and conditions of lives spread across apartments and *bastis*. The forms of surveillance are routinely used for hiring and has quickly become a common tool of control. If technology is commonly considered as providing social levelling, then Lokesh’s account shows how technology-based surveillance is producing new forms of sequestering, control, and inequalities.

Both Wilks and Lokesh point to an interesting pattern in the ways in which efforts at forging a solidarity among servants’ even at a local level tend to be aborted in contemporary times. The implications for our approach towards servants’ collective agency, their negotiating room, and the future of their struggles might be far reaching. It may even signal a need for readjustments in the way the entire issue is to be approached. Wilks has explored the existing fault lines within the ‘class’ of servants along the lines of city-dwellers and commuters which makes the arena of work competitive and contestatory. Lokesh’s interviews with servants reveal how a large number of non-Muslim workers themselves invoked the identitarian categories of ‘Muslims’ or ‘Bangladeshis’ for workers of this religious or regional background. The Muslim workers, they assert, visit their village only once a year, and hence offer to work on lower rates. Conversely, in many places Muslim workers had to hide their identity and resort to using ‘Hindu’ markers such as *sindoor* and *bindi*. Sharma’s earlier work on Delhi suggests that workers invoke the notion of purity alongwith caste and religious identities to differentiate amongst each other. Subject to the force of history, as much as any other social/occupational groups, servants inhabit and perpetuate a hierarchical world in which their own work is situated.^19^ Coupled with the state agencies’ (and even the employers’ occasionally) will to manipulate the caste and community fault lines, this ensures that servants stay internally fractured as a collective. Indeed, they find it difficult even in times of crisis to form fighting solidarities.

The growing informality of the migrant servants’ work, particularly women, has made the work relationship more tenuous and exploitative. Conversely, for some who could speak the English language, domestic work has emerged as a niche for working in expat households in bigger cities such as Delhi.^20^ The larger point is that it is not only the state actors that use ‘divisive’ classificatory categories. The labour market also operates minutely through social and political terms of fractures and segregation – spatial, regional, religious – which affects the desired and a possible solidarity of workers. A migration-based labour market further intensifies and augments the social fissures of class and religion among the domestic workers. ‘Servant hierarchy’ is part of the changing dynamic of master-servant relationship, which should not be addressed as a ‘residue’ of law, labour, state, and capital but as an integral part of it.

In the end, we would like to affirm that our study of domestic servants and servitude is not meant merely to plug historiographic holes and fill the gaps in our knowledge of past. It is not meant merely to pluck relevant information from sources and produce narratives about servants because other historians forgot to do so. Our claims are, for better or worse, far more ambitious. It appears to us that a recalibration of scholarly focus on relations of servitude, especially within the household, (and fully cognizant of the hazy and shifting boundaries of the household itself) can destabilize many historiographic edifices and release new insights not just on the ‘servant issue’ but also on a whole variety of ‘mainstream’ themes as well. It would, however, need a sustained and innovative form of critical engagement with sources, reading them counterintuitively and against the grain. In the contested sphere of Mughal studies, to take but one example, one of the most abiding foci of the more ‘progressive’ historiographic strands has been the category of ‘peasants’ understood mostly as the tax-paying cultivator of land. Peasants apparently were the most exploited of the Mughal subjects, which ironically also meant that the entire state survived primarily on the taxes they paid.^21^ Yet, the category of the Mughal peasant was rarely unpacked to make any functional or social differentiation between the householders, the rich peasants, the poor peasants, and the ‘domestic’ servants who the Sanskrit sources often mention as being ‘owned’ by or ‘attached’ to the ‘householders’, and for whom the brahmanic law books laid out a whole variety of injunctions.^22^ So long as the tax-paying cultivator and householder stood for all of the peasantry, it was easy to see the fault lines primarily as fiscal and existing only between the state and the peasants. Even the various categories of ‘zamindars’ [landlords] figured in this narrative only as trouble makers for the state.

If one was to step back from this historiographic thicket and imagine mapping relations of servitude from the smallest unit of the land-owning peasant household all the way up to the royal household, a much more complex and graded picture of relations of power and regimes of surplus appropriation would be revealed. These complexities cannot be subsumed under debates on nature or centralization of state. Nor can it be substituted by an exploration of Mughal engagements with the multilingual cultures of the subcontinent. Yet, the latter might potentially be used to reap in new sources and to philologically dig into them in ways that will illuminate the lives of both the invisibilized servant in the apparently hapless peasant taxpayers’ home as well as the handsomely paid mansabdars whose fortunes often depended on how convincingly they could profess and practice bandagi (literally slavery) towards their royal master as much as it depended on their precise *mansab* (rank) within the official hierarchy. Unfortunately, we know as little about the largely unwritten codes of conduct for the servants of the Mughal state as we know about the domestic servants of the rural or urban medieval household.

The recalibration of focus that we argue for would also help bring in greater focus on the fate and hold of caste/varna structure during the early modern period. Once we unpack the category of the peasant beyond a simple distribution of property, the inevitable, if difficult, question will emerge: would a poor shudra peasant share the same fate as an equally poor brahmin peasant?

One of the reasons why self-professedly Marxist historians looking for networks of exploitation during the middle ages often missed domestic servitude is because of their jettisoning of the caste as a category powerful enough to have consequences for the processes of production. Yet, the moment one shifts one’s gaze to forms of domestic servitude, and the nature of the (on occasions, nearly infinite) elasticity of the domestic itself, the role of caste/varna order cannot be ignored. If the early modern discourses around servitude often revolved around concerns for loyalty of the servant, whether those of the state or of the household, the colonial (and even the contemporary) archives appear to shift gears, and frame the ‘servants’ problem’ as one of state regulations. Did this index a long-term shift in the ways in which mobilization of and control over labour across the realm of the ‘unproductive’ (read domestic) to the productive (read the artisanal and the industrial) was ensured? We do not have a definite answer to the question, which deserves more sustained exploration than what we have managed. But these questions need to be raised and we hope that future researches in the field we strive to open up will help answer them.

## Notes

1. This is one of the ‘problems’ which lawyers and activists encounter in their engagement with state representatives as the state-driven legal thinking refuses to treat household as a workplace where legal regulation of work can freely operate. Such an ideological recasting of Indian household as an exceptional place has a long history, in which law itself has played a crucial role. See Singha, “Making the Domestic More Domestic”. This exceptionalism was further consolidated in the first half of the nineteenth century when legal debates frequently presented slavery and servitude in Indian households to be ‘benign’ and ‘mild’. See Cassels, *Social Legislation*, 173¯209. Post slavery abolition, in the case of domestic servants, the argument of exceptionalism to keep Indian households outside of the civil mode of regulation persisted throughout the nineteenth and twentieth centuries.

2. For the early beginning of this form of labour market of the domestics and the ways in which the colonial state, drawing upon the essence of British master-servant laws, tried to regulate their work and wages, see Sinha, “Who Is (Not) a Servant, Anyway?”.

3. Steinfeld, *Coercion, Contract, and Free Labour.*

4. Albin, “From ‘Domestic Servant’ to ‘Domestic Worker’”.

5. Ray and Qayum, *Cultures of Servitude.*

6. The most representative writing on this so far is of Neetha and Palriwala, “The Absence of State Law”.

7. The few exceptions are Banerjee, *Men, Women, and Domestics*; Sen and Sengupta, *Domestic Days*.

8. For an earlier exploration covering wide spectrum but without any dedicated focus on domestic servitude, see Patnaik and Dingwaney, eds, *Chains of Servitude*.

9. Sinha, Varma and Jha, *Servants’ Pasts*, Vol. 1; Sinha and Varma, *Servants’ Pasts*, Vol. 2. The present collection of essays emerges from the same project ‘Domestic Servants in Colonial India’ which was funded by European Research Council (ERC-Stg DOS 640627) between 2015 and 2018. The papers collected here were presented in the second international conference of the project organized in April 2018 in Berlin. We thank all the participants and commentators of the conference, especially the contributors to this special issue who share the editors’ conviction about the scholarly value of working on a lesser-developed, if challenging, topic across varied regional settings and temporal zones. In the same vein we also thank all those, particularly Josefine Hoffmann, Nico Putz, and Lilly Kempf from Leibniz-Zentrum Moderner Orient and re:work (Humboldt University) whose planning and labour was indispensable for hosting a three-day long conference. Finally, we thank Ferdinand Schlechta (from ZMO) who painstakingly brought all papers to conform the stylistic standards of the journal.

10. The complexity further grows when we notice that in a particular context of colonial cultural hegemony, *chakri* was perceived as a form of bondage, particularly to the new sensibility of time. Sarkar, ‘“Kaliyuga’, ‘Chakri’ and ‘Bhakti”’.

11. On the ideological premises of service in Mughal India see Rizvi, “Domestic Service in Mughal South Asia”; and Kumar, “Theorising Service with Honour”.

12. Also note the special etiquette on how to eat and how to command the food providers in the genre of Mirzanamas. For instance, Husain, “The Mirza Namah”; and Ahmad, “The British Museum Mizanama”.

13. Also see, Curley ‘“Voluntary’ Relationships and Royal Gifts” along with other essays on robes in Gordon, *Robes of Honour*.

14. For instance, Chughtai’s short story titled *Lihaaf* and Manto’s *Blouse*.

15. Sen and Sengupta, *Domestic Days*, ch. 4.

16. In another ethnographic study from Uttar Pradesh, it has been pointed out that employers call the girls working in their families as ‘kaam karne wali’ (those who perform work), thus prioritizing work as against ‘naukrani’ (maid-servant) which is only used if the employer was angry with the servant. Those recruited through agencies, as Wilks points out, prefer to call themselves ‘centre girls’ thus creating the semblance of the contractual system of hiring as against the social network through which hiring was done in the past. It is worth noting that agency workers do not actually have ‘contracts’. In Kolkata at least, agencies are merely intermediaries. They introduce workers to employers and do not generally provide them with contracts or any other documentary protection. This is one reason that in recent bills on regulation of domestic workers, a great emphasis has been placed on regulating these placement agencies as their role in human trafficking has also come to the light. In regard to terms, ‘helper’, which is a common designate to call servants in urban, metropole India, is not used in small towns, as noted in the research done on the Uttar Pradesh town (in order to keep the identities of the interviewees safe, the authors have given a fictional name ‘Batlipur’ to the town). See Wasiuzzaman and Wells, “Assembling Webs of Support”.

17. Grover, Chambers and Jeffery, “Portraits of Women’s Paid Domestic-Care Labour”.

18. Sharma, “Of Rasoi ka Kaam/Bathroom ka Kaam”.

19. Ibid.

20. Grover, “English-speaking and Educated Female Domestic Workers”.

21. Of the numerous studies, many repetitive, the foundational and the most dominant remains, Habib, *Agrarian System of Mughal India*.

22. See Davis, Jr., “Slaves and slavery in the *Smrticandrika*”. One study that lists and seeks to describe, even if somewhat confusingly, the different types of peasants and different kinds of land relations that existed under the Mughals is of B. R. Grover published in the inaugural issue of the *IESHR* in 1963. See, Grover, “Nature of Land Rights in Mughal India”.
